# *Lactobacillus paracasei* CNCM I-4034 and its culture supernatant modulate *Salmonella*-induced inflammation in a novel transwell co-culture of human intestinal-like dendritic and Caco-2 cells

**DOI:** 10.1186/s12866-015-0408-6

**Published:** 2015-04-01

**Authors:** Miriam Bermudez-Brito, Sergio Muñoz-Quezada, Carolina Gómez-Llorente, Esther Matencio, Fernando Romero, Angel Gil

**Affiliations:** 1 Institute of Nutrition and Food Technology “José Mataix”, Department of Biochemistry and Molecular Biology II, University of Granada, Biomedical Research Center, Avenida del Conocimiento s/n, 18100 Armilla, Granada, Spain; 2 Hero Global Technology Center, Hero Spain, S.A., 30820 Alcantarilla, Murcia Spain

**Keywords:** Caco-2, Cytokines, Dendritic cells, *Lactobacillus*, Gene regulation, Pathogens, Probiotics

## Abstract

**Background:**

The action of probiotics has been studied *in vitro* in cells isolated from both mice and humans, particularly enterocytes (IECs), dendritic cells (DCs) and co-cultures of peripheral DCs and IECs. Peripheral DCs and murine DCs differ from human gut DCs, and to date there are no data on the action of any probiotic on co-cultured human IECs and human intestinal DCs. To address this issue, a novel transwell model was used. Human IECs (Caco-2 cells) grown in the upper chamber of transwell filters were co-cultured with intestinal-like human DCs grown in the basolateral compartment of the transwells. The system was apically exposed for 4 h to live probiotic *L. paracasei* CNCM I-4034 obtained from the faeces of breastfed infants or to its cell-free culture supernatant (CFS) and challenged with *Salmonella typhi*. The secretion of pro- and anti-inflammatory cytokines in the basolateral compartment was determined by immunoassay, and the DC expression pattern of 20 TLR signaling pathway genes was analysed by PCR array.

**Results:**

The presence of the live probiotic alone significantly increased IL-1β, IL-6, IL-8, TGF-β2, RANTES and IP-10 levels and decreased IL-12p40, IL-10, TGF- β1 and MIP-1α levels. This release was correlated with a significant increase in the expression of almost all TLR signaling genes. By contrast, incubation of the co-culture with CFS increased IL-1β, IL-6, TGF-β2 and IP-10 production only when *Salmonella* was present. This induction was correlated with an overall decrease in the expression of all TLR genes except *TLR9*, which was strongly up-regulated.

**Conclusions:**

The data presented here clearly indicate that *L. paracasei* CNCM I-4034 significantly increases the release of pro-inflammatory cytokines, enhances TLR signaling pathway activation and stimulates rather than suppresses the innate immune system. Furthermore, our findings provide evidence that the effects of probiotics in the presence of IECs and DCs differ from the effects of probiotics on cultures of each cell type alone, as reported by us earlier. Thus, co-culture systems such as the one described here are needed to characterise the effects of probiotics *in vitro*, highlighting the potential utility of such co-cultures as a model system.

**Electronic supplementary material:**

The online version of this article (doi:10.1186/s12866-015-0408-6) contains supplementary material, which is available to authorized users.

## Background

Intestinal homeostasis is regulated by tight crosstalk between intestinal epithelial cells (IECs) and immune cells, especially dendritic cells (DCs) [1]. Signaling via innate pattern-recognition receptors (PRRs) such as Toll-like receptors (TLRs), NOD-like receptors (NLRs) and C-type lectin receptors (CLRs) [2] directly influences the chemokine and cytokine response of DCs, as well as the crosstalk between the epithelium and immune cells in the lamina propria [3]. This signaling occurs largely through activation of the transcription nuclear factor kappa B (NF-κB) or mitogen-activated protein kinase (MAPK) pathways [4]. Furthermore, several studies have demonstrated that the typical phenotype of mucosal DCs can be obtained after co-culture with polarised epithelial cells, creating a “tolerogenic” environment [5,6].

Probiotics are live microorganisms that have beneficial effects on the host. Although their mechanism of action is poorly understood [2], they are considered to be promising alternatives to antibiotics for the control and prevention of intestinal infections [7,8]. *Lactobacillus paracasei* CNCM I-4034 is a novel probiotic strain that was obtained from the faeces of breastfed infants and selected based on its probiotic properties, including adhesion to intestinal mucus, sensitivity to antibiotics and resistance to gastrointestinal juices, biliary salts, NaCl and low pH [9]. Previously, we reported that *L. paracasei* culture supernatant inhibits the growth of enterotoxigenic and enteropathogenic bacteria such as *E. coli*, *Salmonella* and *Shigella* [10].

*In vitro* studies of host immune responses have focused on probiotics and enterocytes or DCs. However, single cultures do not reflect the interactions occurring in the intestinal mucosa [11]. Thus, it is important to develop *in vitro* systems that include both DCs and IECs, both of which are crucial for intestinal homeostasis. Using a co-culture model including Caco-2 cells (IECs) and PBMCs, Haller *et al.* reported differential IEC activation by *Escherichia coli* and *Lactobacillus* strains [12]. However, PBMCs and murine DCs are quite different from human DCs [13]. Recently, using human intestinal-like DCs as a model, we reported the ability of *L. paracasei* CNCM I-4034 and its cell-free culture supernatant (CFS) to activate these immune cells [14]. Interestingly, these human DCs are Langerhans-like cells that extend dendritic processes and sample antigens similarly to the lamina propria DCs in the gut that sample luminal antigens [15]. We demonstrated previously that exposure to *Salmonella enterica* serovar *typhi* (*S. typhi*) led to reduced production of pro-inflammatory cytokines and chemokines in the presence of the probiotic and its supernatant. Both enhanced innate immune responses through the activation of TLR signaling pathway [14]. Our results coincide with those of another study indicating that live probiotic bacteria affect the intestinal immune response, whereas secreted components exert anti-inflammatory effects in the gastrointestinal tract [16]. Continue with this line, in the present work we investigated whether *L. paracasei* CNCM I-4034 or its CFS modulates the release of cytokines by intestinal-like DCs through a physical barrier of Caco-2 cells and measured the effects that occur when the system is challenged with *S. typhi*. In addition, the expression pattern of selected TLR cascade signaling genes under these conditions was studied. The novel transwell model described here may better mimic the *in vivo* situation in which beneficial commensal probiotics interact with host immune cells via enterocytes.

## Methods

### Ethic statement

The ethical Committee of Granada’s University approved this study. This study was conducted according to the guidelines laid down in the Declaration of Helsinki and the protocol using human DCs generated from umbilical cord blood CD34^+^ progenitor cells was approved by the Ethical Committee of the University of Granada.

### Design of the study

**Figure 1:**
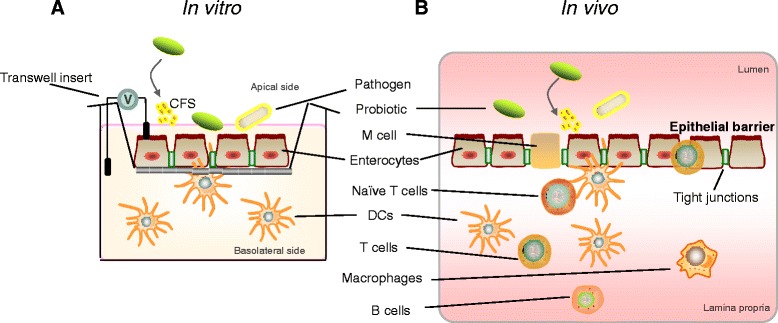
**Design of the study. A** illustrates the *in vitro* transwell model. A monolayer of intestinal epithelial cells (IECs) is formed on a membrane separating two compartments. Human intestinal-like dendritic cells (DCs) are seeded on the lower chamber. **B** is a schematic diagram of the cellular constituents of the intestinal mucosa. CFS: cell-free supernatant of probiotic; V: voltmeter.

A model was developed based on an *in vitro* transwell co-culture of IECs and human intestinal-like DCs. Caco-2 cells were cultured in the upper part of the transwell inserts, with human intestinal-like DCs in the lower chamber. *L. paracasei* CNCM I-4034 and *S. typhi* CECT 725, as well as CFS from *L. paracasei,* were added apically either alone or in combination (Figure 1). Negative control co-cultures were not exposed to any bacteria or CFS. As a positive control of TLR signaling pathway activation *E. coli* lipopolysaccharide (LPS) stimulation was used.

### Bacterial strains and culture conditions

*L. paracasei* CNCM I-4034, which was selected for its immunomodulatory capacity, was isolated from the faeces of breast-fed newborns [9]. The probiotic strain was grown in De Man-Rogosa-Sharpe (MRS) broth medium (Oxoid, Basingstoke, United Kingdom) under anaerobic conditions at 37°C. The bacterial cultures were grown overnight until they reached the stationary phase.

*Salmonella typhi* CECT 725 was provided by the Spanish Type Culture Collection (CECT; Burjassot, Spain). *Salmonella* was cultured for 8 h at 37°C in tryptone soy broth (Panreac Química, Barcelona, Spain) and then subcultured 1:500 in RPMI 1640 medium (Sigma-Aldrich) containing 10% foetal bovine serum (FBS; Gibco Invitrogen, Paisley, United Kingdom) at 37°C overnight.

### Preparation of cell-free culture supernatant of *L. paracasei* CNCM I-4034

The CFS was prepared as described by Bermudez-Brito *et al.* [14]*.* Briefly, the bacteria were grown in MRS broth for 24 h. The *L. paracasei* culture was centrifuged at 12,000 × *g* for 10 min, neutralised to pH 7.0 with 1 N NaOH and concentrated tenfold by lyophilisation. The CFS was filter-sterilised using 0.22-μm pore size filters (Minisart hydrophilic syringe filter; Sartorius Stedim Biotech GmbH, Goettingen, Germany) and stored at −20°C until use. The supernatant was added at 7% v/v.

### Preparation of epithelial cell monolayers

Caco-2 cells were cultured in Dulbecco’s modified Eagle’s medium (DMEM) (Sigma-Aldrich) supplemented with 10% inactivated FBS, 1% glutamine, penicillin G (0.1 U/ml), and streptomycin (0.1 mg/ml). The cells were cultured at 37°C in an atmosphere of 5% CO_2_ and 95% air. Caco-2 cells were seeded in the upper chamber of a transwell filter (3 μm pore size, 6.5 mm diameter; Corning, NY) and incubated for 15–21 days. The cells were grown to confluence (until the trans-epithelial resistance (TER) reached 300 Ωcm^2^).

### Dendritic cells

Human intestinal-like DCs generated from umbilical cord blood CD34^+^ progenitor cells (haematopoietic stem cells) were supplied by MatTek Corporation (Ashland, MA) and maintained according to the supplier’s instructions [15].

### Co-culture of Caco-2 cells and intestinal-like DCs and incubation with *L. paracasei* or its cell-free supernatant and *S. typhi*

Transwell inserts containing adherent monolayers of Caco-2 cells were positioned upside down in a 6-well plate, and 5 × 10^4^ DCs were placed on the membrane. The DCs were allowed to adhere to the filters at 37°C. After 4 h, the transwell inserts were turned right side up and placed in 24-well plates containing 3 × 10^5^ DCs/well in the lower chamber. *L. paracasei* CNCM I-4034 (10^7^ CFU/ ml), its CFS or *S. typhi* CECT 725 (10^6^ CFU/ ml) were added from the apical surface (top chamber), and the cells were incubated for 4 h. Combinations of the probiotic or its CFS with *S. typhi* were prepared similarly. For the incubations, fresh cytokine and antibiotic-containing medium (DC-MM) was replaced with RPMI-1640 medium. After incubation, the bacteria or CFS were washed out of the wells, and the medium was replaced with DC-MM. After 20 h, the culture supernatants were collected from the lower chamber for cytokine analysis, and the DCs in that compartment were collected for RNA extraction.

LPS at 20 ng/ml (Sigma-Aldrich) was used as a positive control. Negative control cultures contained unstimulated co-cultured DCs and Caco-2 cells.

### Cytokine and chemokine quantification in culture supernatants

IL-1β, IL-6, IL-8, IL-10, IL-12 (p40), IL-12 (p70), TNF-α, IFN-γ, MCP-1/CCL2, MIP-1α/CCL3, RANTES/CCL5, MDC/CCL22, IP-10/CXCL10 and TGF-β were measured by immunoassay with a MILLIplex™ kit (Linco Research Inc, Missouri, USA) using the Luminex 200 system according to the manufacturer’s instructions. Three independent experiments were performed.

### Extraction of RNA from DCs and evaluation of the expression of selected genes of the TLR signaling pathway

Briefly, DCs collected from the lower chamber were lysed, and total RNA was extracted using the RNAqueous Kit (Ambion, Paisley, United Kingdom) and Turbo DNase treatment (Ambion) according to the manufacturers’ recommendations. The quality of the RNA was verified on a Model 2100 Bioanalyzer (Agilent, Santa Clara, USA), and the RNA concentration was determined using a Rediplate 96 Ribogreen RNA Quantitation Kit (Gibco Invitrogen). Three samples were taken per treatment.

Real time RT-PCR analysis was carried out using a Human TLR Signaling Pathway PCR Array (SABiosciences Corporation, Frederick, Maryland, USA), which includes primer pairs specific for 20 selected genes related to TLR-mediated signaling pathways, as previously described by Bermudez-Brito *et al.* [14]. The expression level of each gene was analysed with RT_2_ Profiler PCR Array Data Analysis software (version 3.4; SABiosciences). The change in expression of each gene was reported as the fold change in expression (Fc). The results reflect logarithmic fold increase relative to the control samples.

Finally, IECs collected from the transwell membrane were lysed, and total RNA was extracted using the same method as described above. Real time RT-PCR analysis was carried out using a Human TLR Signaling Pathway PCR Array. The results are provided as [Additional files 1, 2, 3 and 4].

### Statistical analysis

All data are expressed as the mean ± standard error of the mean (SEM) of three independent experiments. A two-sided Mann–Whitney *U*-test was used to determine changes in cytokine release and Fc gene differences between treatments. Statistical calculations were performed using NCSS 2007 software (Kaysville, UT). Differences between the treated cells and the controls were considered statistically significant when the P values were less than 0.05 (*).

## Results

### Live *L. paracasei* CNCM I −4034 is a potent inducer of cytokine production

**Figure 2:**
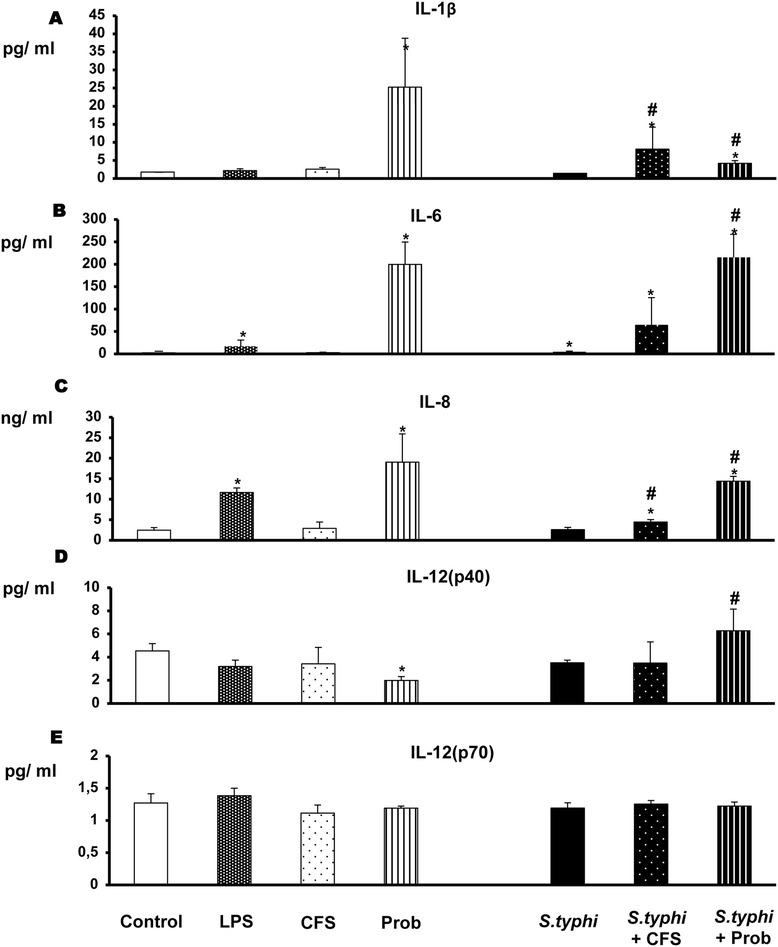
**Effects of** ***L. paracasei*** **CNCM I-4034 and its cell-free culture supernatant on the secretion of pro-inflammatory cytokines by intestinal-like human dendritic cells through an epithelial layer.** The production of IL-1β **(Panel A)**, IL-6 **(Panel B)**, IL-8 **(Panel C)**, IL-12 (p40) **(Panel D)** and IL-12 (p70) **(Panel E)** was measured. DCs and Caco-2 cells were incubated for 4 h with the live probiotic *L. paracasei* or its supernatant, *Salmonella*, or both and further incubated for 20 h in medium containing antibiotics. The culture supernatants were collected from the lower chamber, and the cytokine levels in the supernatants were determined by immunoassay. The data shown are the mean and SEM of the values obtained in three different experiments. *, p<0.05 compared with controls; #, p<0.05 compared with S.typhi.

**Figure 3:**
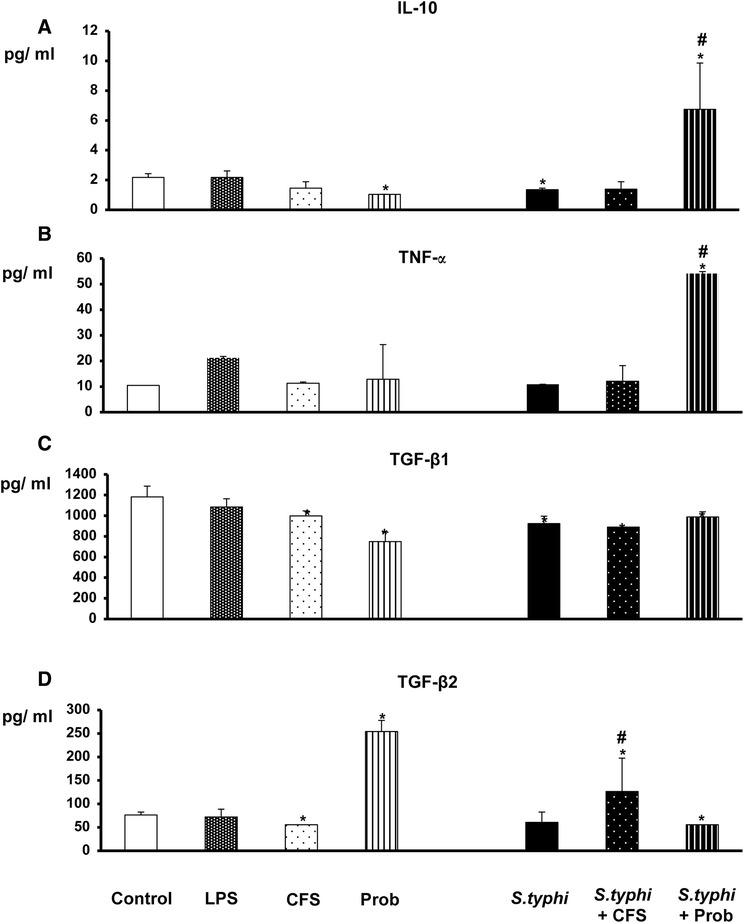
**Measurement of anti-inflammatory cytokines and TNF-α in DCs through an epithelial layer after exposure to** ***L. paracasei*** **,** ***Salmonella*** **or a combination of the two.** The production of IL-10 **(Panel A)**, TNF-α **(Panel B)**, TGF-β1 **(Panel C)** and TGF-β2 **(Panel D)** was measured. DCs and Caco-2 cells were incubated for 4 h with the live probiotic *L. paracasei* or its supernatant, *Salmonella* or both and further incubated for 20 h in medium containing antibiotics. The culture supernatants were collected from the lower chamber, and the cytokine levels were determined by immunoassay. The data shown are the mean and SEM of the values obtained in three different experiments. *, p<0.05 compared with controls; #, p<0.05 compared with S.typhi; N.D. indicates not detected.

**Figure 4:**
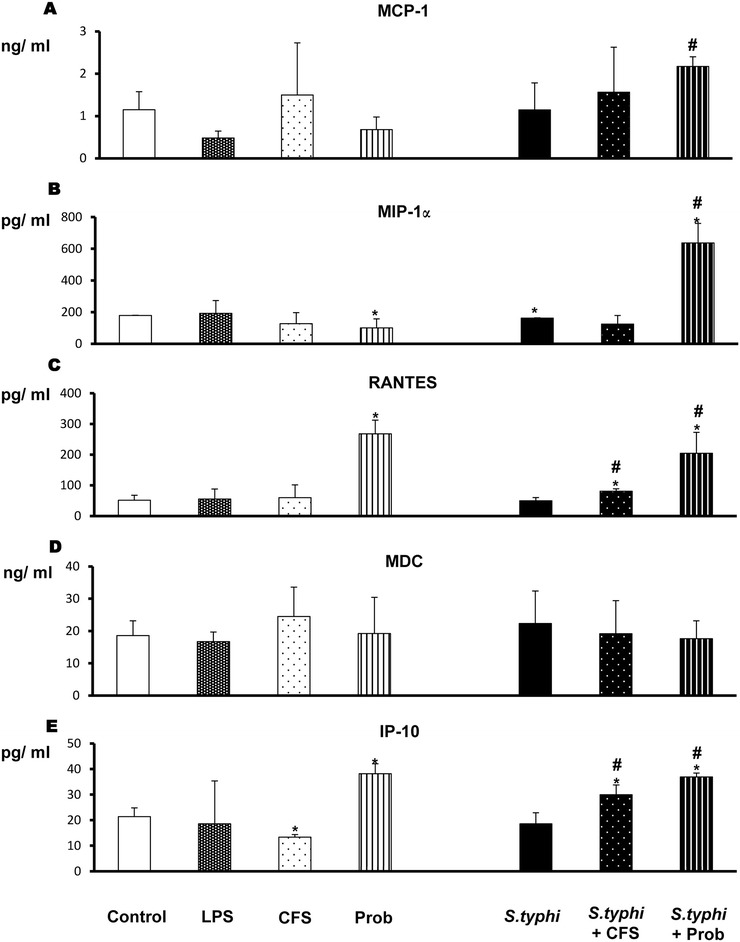
**Measurement of chemokines in DCs through an epithelial layer after exposure to** ***L. paracasei*** **,** ***Salmonella*** **or a combination of the two.** The production of MCP-1/CCL2 **(Panel A)**, MIP-1α/CCL3 **(Panel B)**, RANTES/CCL5 **(Panel C)**, MDC/CCL22 **(Panel D)** and IP-10/CXCL10 **(Panel E)** was measured. DCs and Caco-2 cells were incubated for 4 h with the live probiotic *L. paracasei* or its supernatant, *Salmonella* or both and further incubated for 20 h in medium containing antibiotics. The culture supernatants were collected, and the cytokine levels were determined by immunoassay. The data shown are the mean and SEM of the values obtained in three different experiments. *, p<0.05 compared with controls; #, p<0.05 compared with S.typhi; N.D. indicates not detected.

We first studied the effects of the probiotic strain and its CFS on cytokine and chemokine production in co-cultures of human DCs and IECs. As shown in Figures 2, 3 and 4, we found that live *L. paracasei* CNCM I-4034 was a potent inducer of the pro-inflammatory cytokines IL-1β and IL-6 and the chemokine IL-8. In addition, we found that *L. paracasei* CNCM I-4034 increased the production of TGF-β2, RANTES/CCL5 and IP-10/CXCL10. In contrast, it decreased IL-12p40 (Figure 2), IL-10, TGF- β1 (Figure 3) and MIP-1α (Figure 4) levels but had no effect on IL-12p70 (Figure 2) levels or on the levels of any of the other chemokines tested (Figure 4).

As expected, LPS stimulation (the positive control) induced strong production of IL-6 and IL-8 (Figure 2). TNF-α was also induced; however, the difference never reached statistical significance (Figure 3). LPS did not induce chemokine release (Figure 4).

When we analysed the cytokines produced by co-cultures exposed to *L. paracasei* CFS, we found that the cells were not very responsive to the CFS (Figures 2, 3 and 4). CFS-treated co-cultures exhibited a cytokine and chemokine profile similar to that of negative controls (non-exposed co-cultures) but showed less induction of TGF-β1 and β2 (Figure 3) and IP-10 (Figure 4).

### Live probiotic *L. paracasei* notably increases the release of cytokines and chemokines in the presence of *Salmonella*

With respect to cytokine production, incubation of the co-culture with *Salmonella* alone did not increase the secretion of pro-inflammatory cytokines or chemokines (Figures 2, 3 and 4); only IL-6, IL-10, TGF-β1 and MIP-1α levels were slightly modified.

As shown in Figures 2, 3 and 4, co-treatment with live *L. paracasei* CNCM I-4034 and *Salmonella* induced the release of a number of cytokines such as IL-6 and TNF-α and of chemokines such as MIP-1α/CCL3 and RANTES/CCL5; however, it induced less TGF-β2. Furthermore, this treatment increased IL-10 production. No effects on MCP-1/CCL2, MDC/CCL22, TGF-β1 or IL12p70 levels were observed.

In contrast, incubation with *L. paracasei* CFS and *S. typhi* resulted in increased release of the pro-inflammatory cytokines IL-1β, IL-6 and IL-8 (Figure 2), TGF-β2 (Figure 3), RANTES/CCL5 and IP-10/CXCL10 production (Figure 4) and decreased TGF-β1 production (Figure 3).

### *L. paracasei* and its supernatant induce distinct strain-specific expression profiles of TLR signaling pathway molecules in human DCs

**Figure 5:**
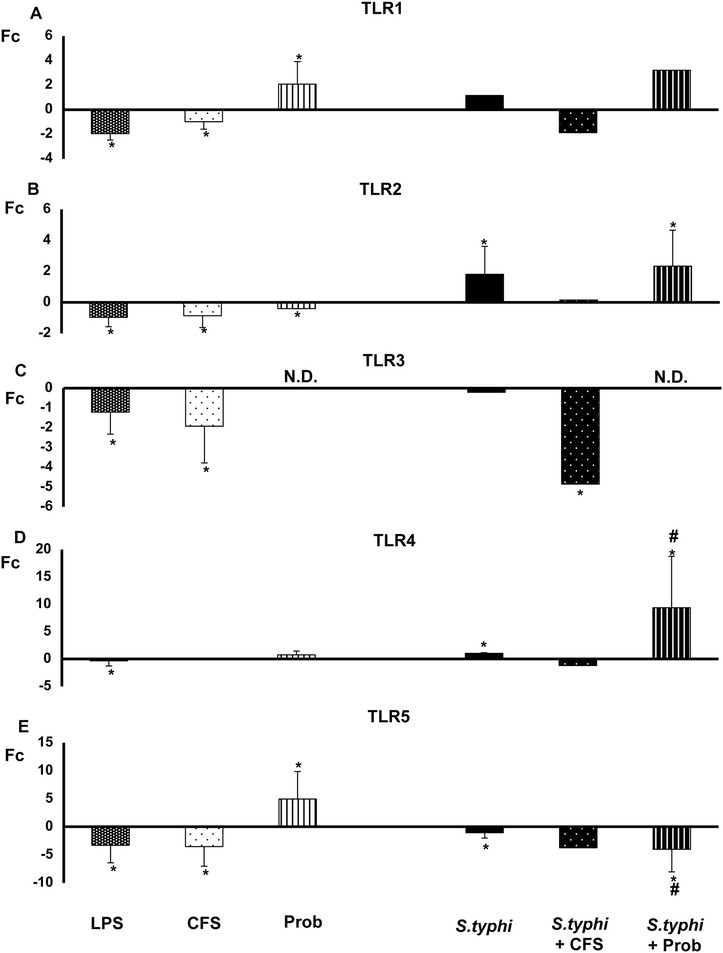
**Expression of** ***TLR*** **genes in DCs in the presence of** ***L. paracasei*** **,** ***Salmonella*** **or a combination of the two.** Comparison of the expression of *TLR1* **(Panel A)** *, TLR2* **(Panel B)** *, TLR3* **(Panel C)** *, TLR4* **(Panel D)** and *TLR5* **(Panel E)** in dendritic cells (DCs) taken from the lower chamber of a transwell co-culture model in the presence of the live probiotic *L. paracasei* or its supernatant, *Salmonella* or both. The fold change (Fc) represents the ratio of the expression in the treated DCs to that of expression in the control cells. *, p<0.05 compared with controls; #, p<0.05 compared with S.typhi; N.D. indicates not detected.

**Figure 6:**
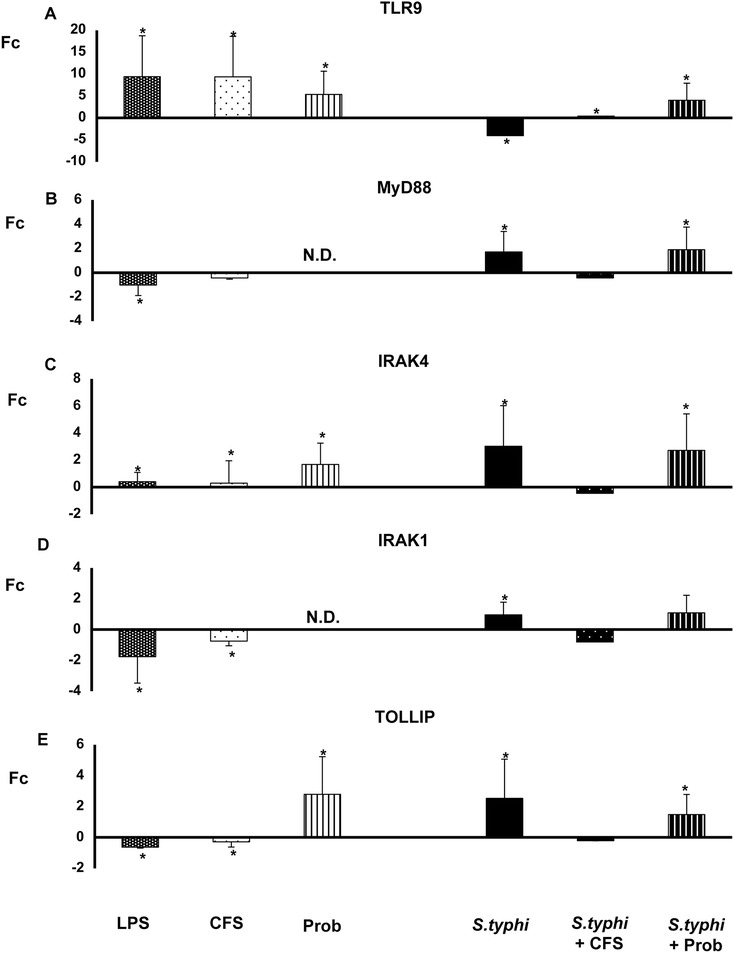
**Expression of TLR signaling pathway components in DCs treated with** ***L. paracasei*** **,** ***Salmonella*** **or a combination of the two.** Comparison of the expression of *TLR9* **(Panel A)** *, MYD88* **(Panel B)** *, IRAK-1* **(Panel C)** *, IRAK-4* **(Panel D)** and *TOLLIP* **(Panel E)** in dendritic cells (DCs) taken from the lower chamber of a transwell co-culture model in the presence of the live probiotic *L. paracasei* or its supernatant, *Salmonella* or both. The fold change (Fc) represents the ratio of the expression in the treated DCs to that of expression in the control cells. *, p<0.05 compared with controls; #, p<0.05 compared with S.typhi; N.D. indicates not detected.

**Figure 7:**
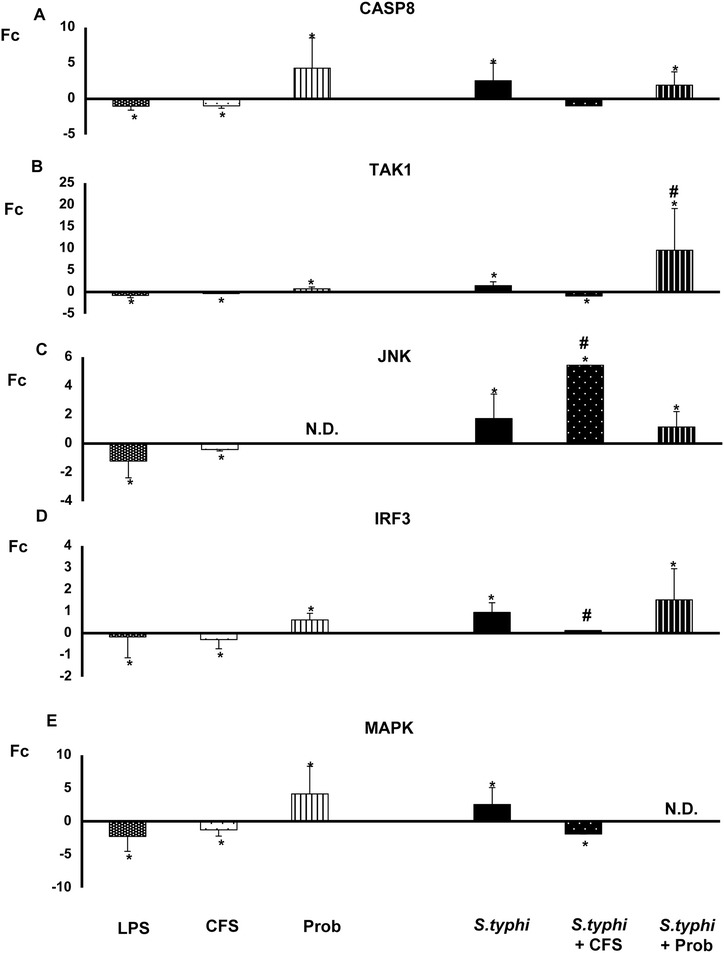
**Expression of TLR signaling pathway components in DCs treated with** ***L. paracasei*** **,** ***Salmonella*** **or a combination of the two.** Comparison of the expression of *CASP8* **(Panel A)** *, TAK-1* **(Panel B)** *, JNK* **(Panel C)** *, IRF-3* **(Panel D)** and *MAPK14* **(Panel E)** in dendritic cells (DCs) taken from the lower chamber of a transwell co-culture model in the presence of the live probiotic *L. paracasei* or its supernatant, *Salmonella* or both. The fold change (Fc) represents the ratio of the expression in the treated DCs to that of expression in the control cells. *, p<0.05 compared with controls; #, p<0.05 compared with S.typhi; N.D. indicates not detected.

**Figure 8:**
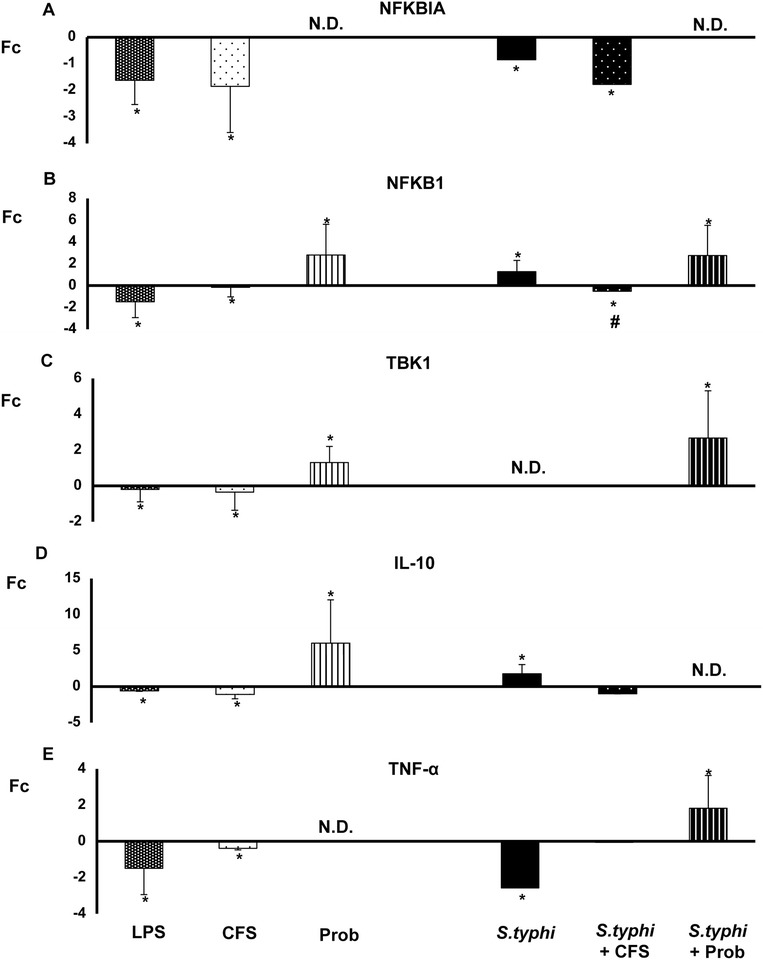
**Expression of TLR signaling pathway components in DCs treated with** ***L. paracasei*** **,** ***Salmonella*** **or a combination of the two.** Comparison of the expression of *NFKBIA* **(Panel A)** *, NFKB-1* **(Panel B)** *, TBK-1* **(Panel C)** *, IL-10* **(Panel D)** and *TNF-*α **(Panel E)** in dendritic cells (DCs) taken from the lower chamber of a transwell co-culture model in the presence of the live probiotic *L. paracasei* or its supernatant, *Salmonella* or both. The fold change (Fc) represents the ratio of the expression in the treated DCs to that of expression in the control cells. *, p<0.05 compared with controls; #, p<0.05 compared with S.typhi; N.D. indicates not detected.

Remarkably, most of the TLR signaling pathway genes tested in DCs were up-regulated upon incubation of the co-culture with *L. paracasei* CNCM I-4034 alone (Figures 5, 6, 7 and 8). The probiotic strain augmented *TLR1*, *TLR4*, *TLR5* and *TLR9* gene expression (Figures 5 and 6). In addition, the live probiotic induced up-regulation of *TOLLIP* (Figure 6), *CASP8* (Figure 7) and *IL-10* (Figure 8).

In contrast, CFS alone inhibited the expression of all DC TLR-selected genes except *TLR9,* which was up-regulated, and *IRAK4*, the expression of which was slightly increased (Figures 5 and 6).

When DCs and Caco-2 cells were co-cultured with live probiotic *L. paracasei* and *Salmonella*, strong and sustained transcription of most of the genes associated with TLR signaling pathways was observed in the human DCs (Figures 5, 6, 7 and 8)*. TLR1*, *TLR2*, *TLR4* and *TLR9* gene expression was increased. Interestingly, in the presence of the enteropathogen, exposure to the live probiotic up-regulated *MYD88* (Figure 6), *CASP8* (Figure 7), *NFKB1* and *TBK1* (Figure 8) as well as other genes. Remarkably, the presence of the probiotic strain and *Salmonella* induced strong up-regulation of *TAK1* (Figure 7).

Interestingly, simultaneous incubation of the co-culture with *L. paracasei* CFS and *Salmonella* exerted a similar effect on TLR gene expression to that of CFS alone except for *JNK* expression, which was strongly up-regulated (Figure 7). Both CFS alone and CFS and *Salmonella* treatments decreased *NFKBIA* gene expression (Figure 8).

Analysis of TLR signaling pathway genes of Caco-2 cells collected from the transwell membrane is provided as Additional files 1, 2, 3 and 4.

## Discussion

To the best of our knowledge, this is the first study in which the immune response elicited by probiotics has been analysed using an *in vitro* bilayer system that includes human IECs growing on transwell membranes and human intestinal-like DCs developed from CD34+ progenitor cells isolated from umbilical cord blood. Moreover, in an attempt to mimic the *in vivo* conditions under which DCs are able to open tight junctions between adjacent epithelial cells and take up bacteria directly from the intestinal lumen [17], the transwell inserts were inverted and the human intestinal-like DCs were allowed to adhere to the IECs for 4 h [18,19]. This culture system will facilitate functional studies, including studies of host-microbe interactions.

Live *L. paracasei* CNCM I-4034 alone was a potent inducer of IL-1β, IL-6 and IL-8. Moreover, in the presence of *Salmonella*, *L. paracasei* induced the production of almost all the cytokines analysed including IL-6, IL-8 and TNF-α, which are regulated by NF-κB [19], conversely to the results reported by our group in single cultures, either in Caco-2 cells [20] or in DCs [14], in which the probiotic *L. paracasei* down-regulated a broad array of pro-inflammatory cytokines in the presence of *Salmonella.* Altogether, the results suggest that *L. paracasei* enhances innate immunity via NF-κB activation rather than suppression. Furthermore, the stimulation of other innate immunity pathways, i.e., ERK mediated IL-10 production and TGF-β production mediated by P38 MAPK, ERK, and JNK, could also be involved [21,22]. This activation of innate immunity may help strengthen intestinal defence and epithelial barrier function.

TGF-β is considered a key immunoregulatory factor in promoting immunoglobulin A (IgA) production [23] and maintaining immune tolerance [24,25]. In this context, live *L. paracasei* stimulated TGF-β2 release. Furthermore, CFS is a potent inducer of TGF-β2 in response to *S. typhi*. Immunomodulatory effects of TGF-β2 have been reported in conditions such as atopy and systemic inflammatory response syndrome [26,27]. In addition, IL-6 was notably secreted upon stimulation of cells with live *L. paracasei* alone or with *L. paracasei* and its CFS in the presence of *Salmonella.* Probiotic strains have been described as potent inducers of IL-6 [28] and enhancers of IgA responses [29]. Uematsu *et al.* [30] proposed that commensal bacteria induce IgA production through a mechanism mediated by TLR5, an interpretation that is consistent with our results. Altogether, these results suggest that *L.paracasei* could lead to the generation of IgA and reinforce epithelial barrier function because secreted IgA limits bacterial association with the intestinal epithelial surface and restricts the penetration of pathogens across the gut epithelium [31], precluding a systemic immune response [32]. Interestingly, this argument is strengthened by our recent observation that *Bifidobacterium breve* CNCM I-4035 administration increases secretory IgA content after 30-day intervention in healthy volunteers [33].

In contrast, IECs and DCs were poorly responsive to *S. typhi*, contrary to the results reported by Zoumpopoulou *et al.* [1] and Vossenkämper *et al.* [19]. In addition, *S. typhi* was unable to induce the release of chemokines such as RANTES and induced low levels of MIP-1α. However, it is important to note that *Salmonella* did induce changes in the expression of TLR signaling pathway genes in human DCs. These changes in gene expression may be due to crossing of the monolayer by bacterial compounds or to direct contact between the pathogen and DCs that crept between the epithelial cells and contacted the pathogen directly from the luminal side. Taken together, our results suggest that *Salmonella* cells were not able to penetrate the epithelial layer and interact directly with the DCs. In addition, TLR genes expression in IECs was decreased in response of *Salmonella* stimuli [see Additional file 1]. Furthermore, *Salmonella* just induced the expression of *MyD88*, *Casp8*, *TAK1* and *NFKBIA* in IECs [see Additional files 1, 2, 3 and 4]. Thus, the low cytokine profile observed in the co-cultured DCs and IECs could be due to the action of IECs and bacterial compounds that crossed the monolayer. Consequently, IECs not only form a physical barrier to bacteria but also regulate DC cytokine production, possibly through epithelial cell-derived factors [34,35], and could contribute to prevention of an exaggerated inflammation and thereby to the maintenance of immune homeostasis in the gut [36].

Interestingly, the culture supernatant exhibited activity when *Salmonella* was present, in agreement with our previous findings [14]. These data indicate that soluble bacterial product(s) released by *L. paracasei* CNCM I-4034 were able to cross the epithelial monolayer and activate the DCs in the lower chamber. Moreover, IECs and DCs are able to recognise probiotic secreted factor(s) without inducing inflammatory reactions [35]. This culture supernatant modulated the ability of the DCs to produce inflammatory cytokines in response to pathogens.

Previous results obtained by our group suggest that probiotics modulate the cytokine profile in immune cells through TLRs stimulation [14]. We therefore analysed the expression of TLRs in DCs taken from the lower chamber of our co-culture system. The first of these experiments showed that few genes were up-regulated by the supernatant in DCs. Exposure to CFS led to an overall decrease in expression of all the TLR genes except *TLR9*, which was strongly up-regulated. This up-regulation was also found in our previous study in immune cells. Conversely, the expression of this TLR was decreased in IECs. Moreover, the expression of *TLR1*, *TLR3* and *TLR4* was up-regulated upon CFS stimulation [see Additional files 1 and 2]. Because CFS by itself did not induce cytokine production, TLR9 may have a key role in the context of probiotic-host interactions, and the results suggest that the immunomodulatory effects of *L. paracasei* product(s) may involve mechanisms that overlap with those associated with CpG [14,2]. Moreover, down-regulation of TLRs genes may block the production of pro-inflammatory cytokines. Interestingly, *JNK* gene expression in DCs was notably increased with this treatment, whereas in IECs was down-regulated [see Additional file 3]. JNK phosphorylation leads to the activation of transcription factors such as NF-κB, which directs the production of pro-inflammatory cytokines [37], consistent with our results. Overall, it appears that in the presence of infectious agents, the supernatant of *L. paracasei* promotes cytokine production and thereby enables the cells to combat pathogens.

In the presence of *Salmonella* and the live probiotic*, TLR2* and *TLR9* were slightly up-regulated in DCs, whereas *TLR1, TLR2, TLR3* and *TLR*4 were increased in IECs. Like the results of several previous studies, our results suggest that exposure to live *L. paracasei* increases the expression of *TLR2* and *TLR9*, activating innate immunity in immune cells [38-40]. Furthermore, TLR2 stimulation appears to enhance the epithelial barrier, and it has been shown that activation of this TLR plays an essential role in resistance to bacterial invasion. Several studies have described increased expression of *TLR9* after the administration of probiotics such as *L. johnsonii* [41]. In this line, and according with our results, it has been shown a protective role for TLR1 during bacterial infection via its signaling in the intestinal epithelium [42]. TLR4 was also up-regulated in our experiments in DCs and IECs. TLR4 can recognise LPS present in the walls of Gram-negative bacteria [43] and plays a key role in defence against *Salmonella* [30]. In the presence of *Salmonella*, TNF-α gene expression is up-regulated, a finding that correlates with the observed cytokine profile. Altogether, these results suggest that when pathogens are present, *L. paracasei* promotes the local stimulation of innate immune responses in the gut through the activation of NF-κB, thus preventing systemic inflammation. Follow-up studies are ongoing to evaluate whether this probiotic strain can exert a protective effect *in vivo* in a murine model.

## Conclusions

This study demonstrates that the effects of probiotics should be studied on complex of cells because cultures of single cell types do not exhibit the effects of the exposure to a probiotic strain. DCs react differently to probiotics and pathogens in the presence and absence of IECs. Co-culture models such as the one presented here will facilitate the study of host-microbe interactions. Moreover, this system also provides a feasible platform for the development of novel probiotics or other agents for further animal studies and clinical trials.

## Additional files

Additional file 1: Figure S1. Expression of *TLR* genes in IECs in the presence of *L. paracasei*, *Salmonella* or a combination of the two. Comparison of the expression of *TLR1* (Panel A)*, TLR2* (Panel B)*, TLR3* (Panel C)*, TLR4* (Panel D) and *TLR5* (Panel E) in Caco-2 cells (IECs) taken from the transwell membrane of a co-culture model in the presence of the live probiotic *L. paracasei* or its supernatant, *Salmonella* or both. The fold change (Fc) represents the ratio of the expression in the treated IECs to that of expression in the control cells. 
Additional file 2: Figure S2. Expression of TLR signaling pathway components in IECs treated with *L. paracasei*, *Salmonella* or a combination of the two. Comparison of the expression of *TLR9* (Panel A)*, MYD88* (Panel B)*, IRAK-1* (Panel C)*, IRAK-4* (Panel D) and *TOLLIP* (Panel E) in Caco 2-cells (IECs) taken from the transwell membrane of a transwell co-culture model in the presence of the live probiotic *L. paracasei* or its supernatant, *Salmonella* or both. The fold change (Fc) represents the ratio of the expression in the treated IECs to that of expression in the control cells. 
Additional file 3: Figure S3. Expression of TLR signaling pathway components in IECs treated with *L. paracasei*, *Salmonella* or a combination of the two. Comparison of the expression of *CASP8* (Panel A)*, TAK-1* (Panel B)*, JNK* (Panel C)*, IRF-3* (Panel D) and *MAPK14* (Panel E) in Caco-2 cells (IECs) taken from the transwell membrane of a transwell co-culture model in the presence of the live probiotic *L. paracasei* or its supernatant, *Salmonella* or both. The fold change (Fc) represents the ratio of the expression in the treated IECs to that of expression in the control cells. 
Additional file 4: Figure S4. Expression of TLR signaling pathway components in IECs treated with *L. paracasei*, *Salmonella* or a combination of the two. Comparison of the expression of *NFKBIA* (Panel A)*, NFKB-1* (Panel B)*, TBK-1* (Panel C)*, IL-10* (Panel D) and *TNF-*α (Panel E) in Caco-2 cells (IECs) taken from the transwell membrane of a transwell co-culture model in the presence of the live probiotic *L. paracasei* or its supernatant, *Salmonella* or both. The fold change (Fc) represents the ratio of the expression in the treated IECs to that of expression in the control cells.
