# Supplementary figures and images for: Lactobacillus paracasei CNCM I-4034 and its culture supernatant modulate Salmonella-induced inflammation in a novel transwell co-culture of human intestinal-like dendritic and Caco-2 cells
Source: BMC Microbiol. 2015 Apr 1;15:79. doi: 10.1186/s12866-015-0408-6 (PMC5353866; doi:10.1186/s12866-015-0408-6)

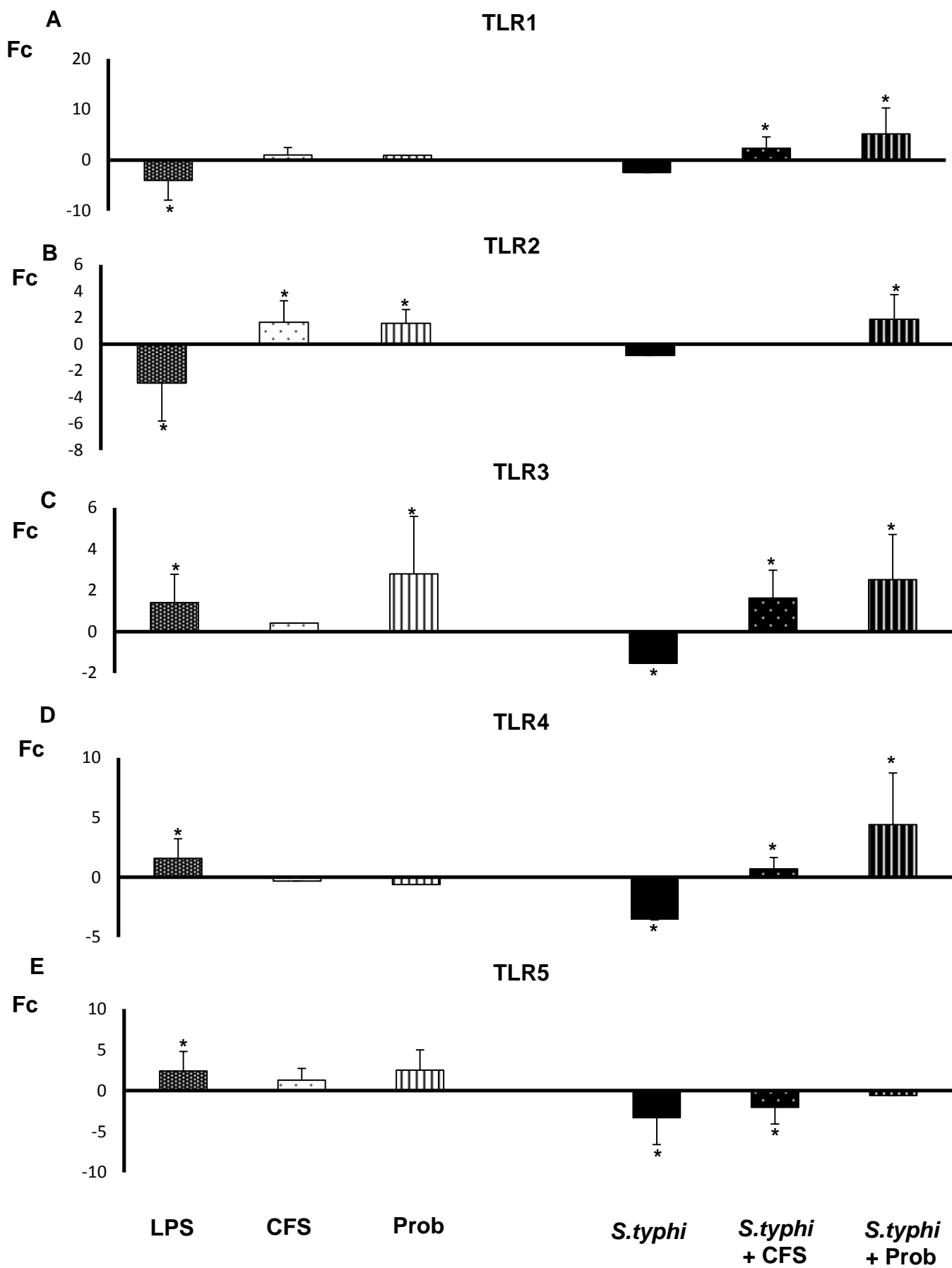

Supplement: Additional file 1: Figure S1. — Expression of TLR genes in IECs in the presence of L. paracasei, Salmonella or a combination of the two. Comparison of the expression of TLR1 (Panel A), TLR2 (Panel B), TLR3 (Panel C), TLR4 (Panel D) and TLR5 (Panel E) in Caco-2 cells (IECs) taken from the transwell membrane of a co-culture model in the presence of the live probiotic L. paracasei or its supernatant, Salmonella or both. The fold change (Fc) represents the ratio of the expression in the treated IECs to that of expression in the control cells. [file 12866_2015_408_MOESM1_ESM.pdf]

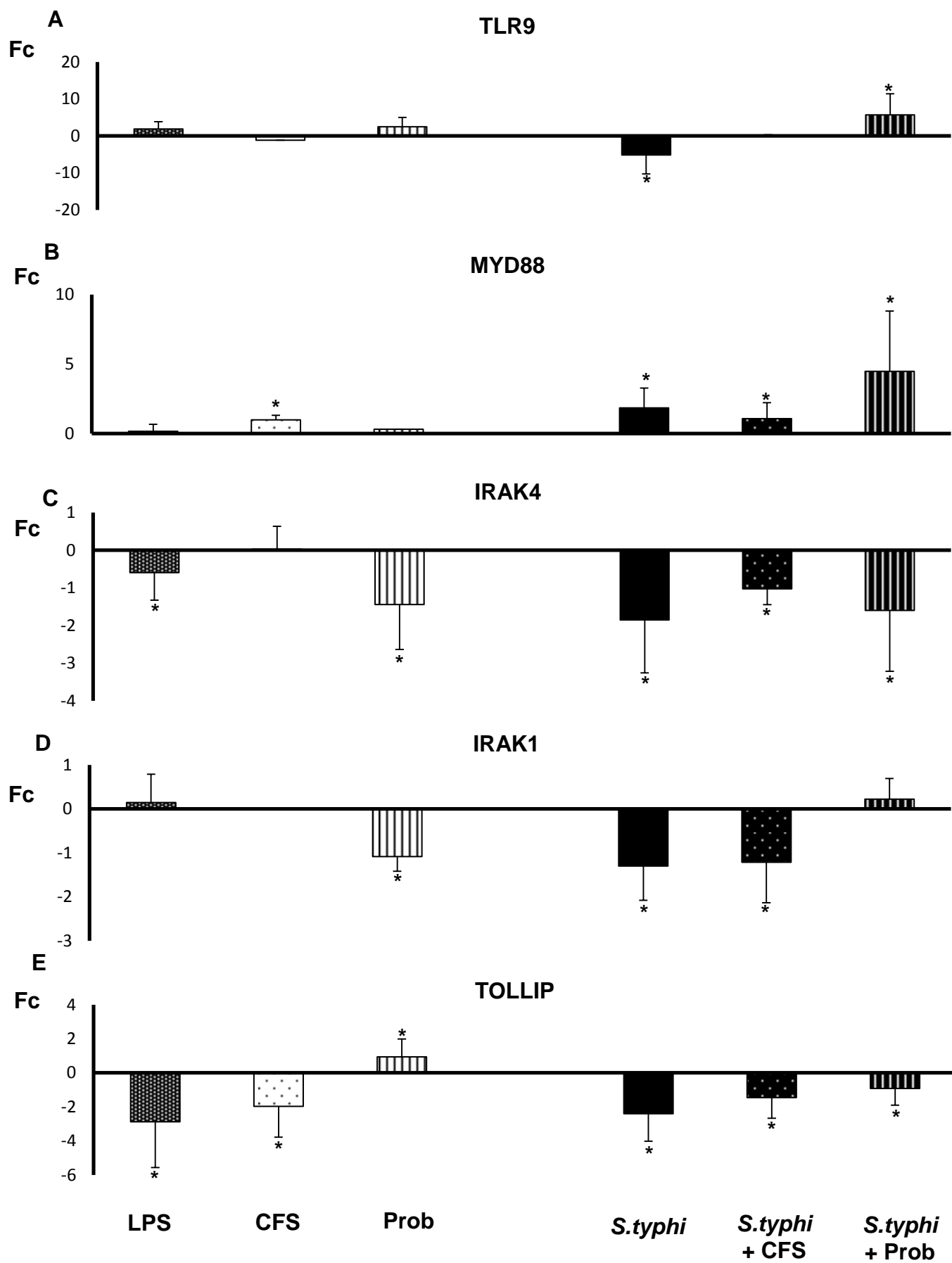

Supplement: Additional file 2: Figure S2. — Expression of TLR signaling pathway components in IECs treated with L. paracasei, Salmonella or a combination of the two. Comparison of the expression of TLR9 (Panel A), MYD88 (Panel B), IRAK-1 (Panel C), IRAK-4 (Panel D) and TOLLIP (Panel E) in Caco 2-cells (IECs) taken from the transwell membrane of a transwell co-culture model in the presence of the live probiotic L. paracasei or its supernatant, Salmonella or both. The fold change (Fc) represents the ratio of the expression in the treated IECs to that of expression in the control cells. [file 12866_2015_408_MOESM2_ESM.pdf]

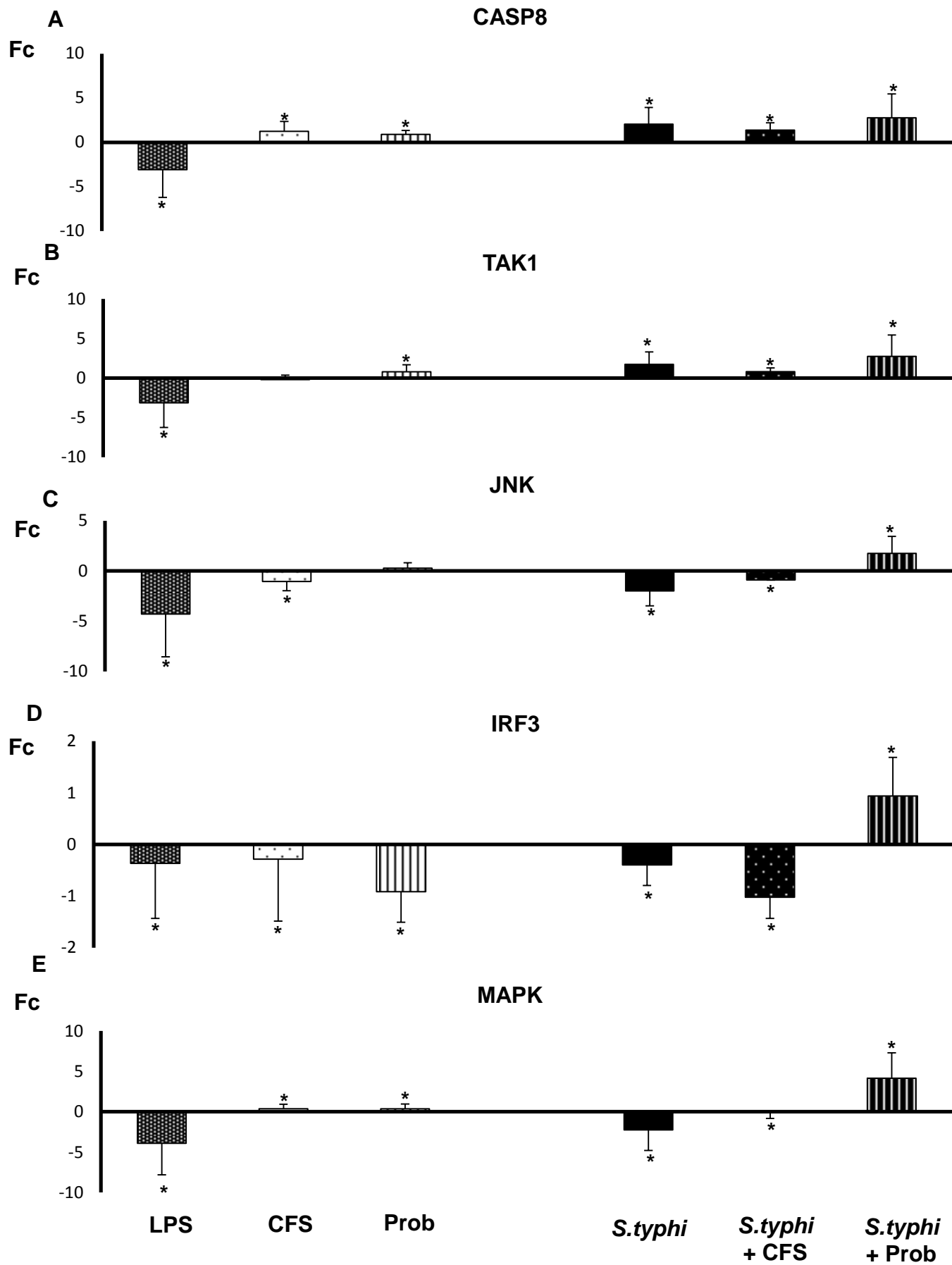

Supplement: Additional file 3: Figure S3. — Expression of TLR signaling pathway components in IECs treated with L. paracasei, Salmonella or a combination of the two. Comparison of the expression of CASP8 (Panel A), TAK-1 (Panel B), JNK (Panel C), IRF-3 (Panel D) and MAPK14 (Panel E) in Caco-2 cells (IECs) taken from the transwell membrane of a transwell co-culture model in the presence of the live probiotic L. paracasei or its supernatant, Salmonella or both. The fold change (Fc) represents the ratio of the expression in the treated IECs to that of expression in the control cells. [file 12866_2015_408_MOESM3_ESM.pdf]

# NFKBIA

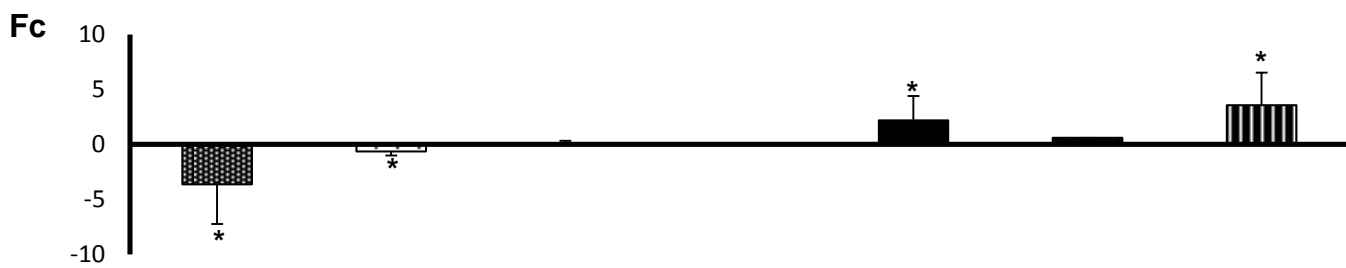

# NFKB1

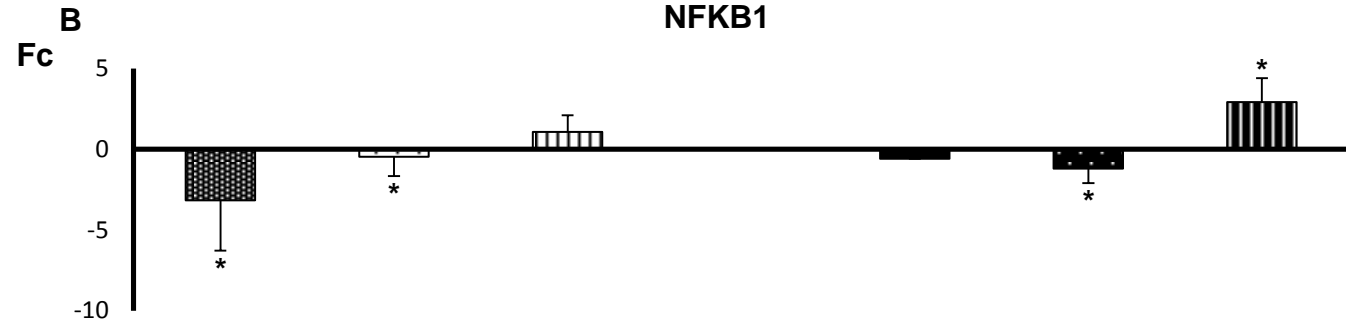

# TBK1

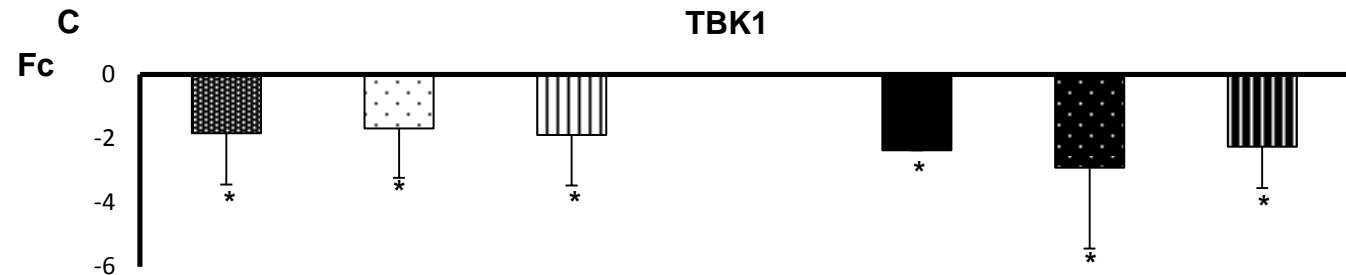

# IL-10

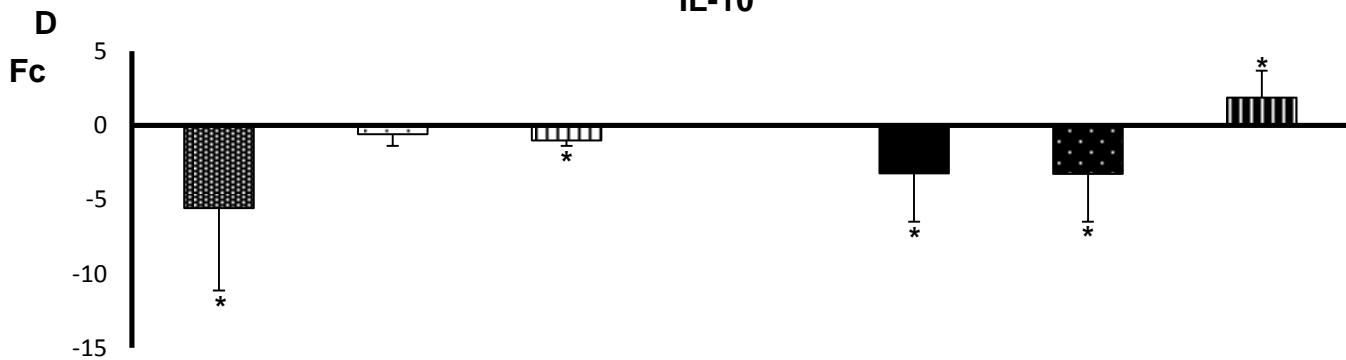

# TNF-α

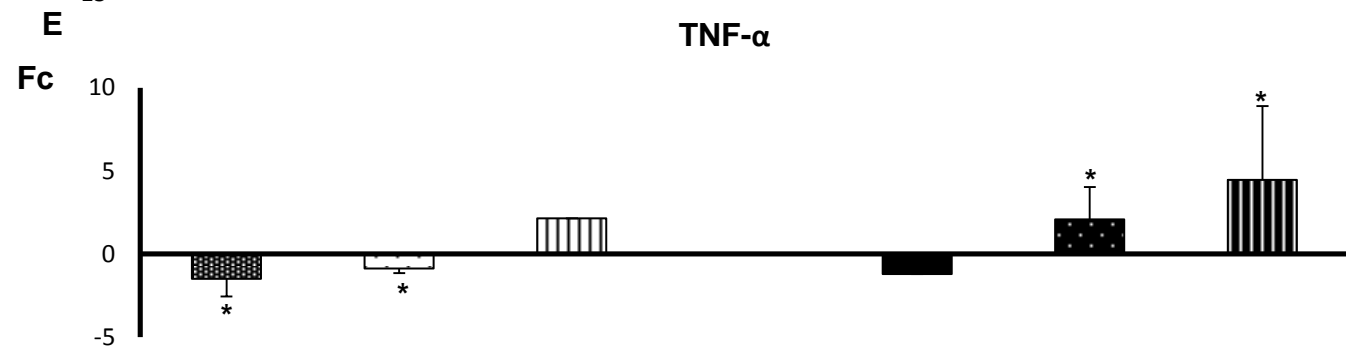

LPS

CFS

Prob

*S.typhi*

*S.typhi*  
+ CFS

*S.typhi*  
+ Prob

Supplement: Additional file 4: Figure S4. — Expression of TLR signaling pathway components in IECs treated with L. paracasei, Salmonella or a combination of the two. Comparison of the expression of NFKBIA (Panel A), NFKB-1 (Panel B), TBK-1 (Panel C), IL-10 (Panel D) and TNF-α (Panel E) in Caco-2 cells (IECs) taken from the transwell membrane of a transwell co-culture model in the presence of the live probiotic L. paracasei or its supernatant, Salmonella or both. The fold change (Fc) represents the ratio of the expression in the treated IECs to that of expression in the control cells. [file 12866_2015_408_MOESM4_ESM.pdf]
